# Strengthening Collaborations for Geriatrics Knowledge: Twitter Journal Club for Geriatric Fellows

**DOI:** 10.1093/geroni/igab046.2816

**Published:** 2021-12-17

**Authors:** Gunjan Manocha, Casey Morton, Elizabeth Chapman, Daniel Pellecer, Kahli Zeitlow, Tanjeev Kaur, Donald Jurivich

**Affiliations:** 1 University of North Dakota, Grand Forks, North Dakota, United States; 2 University of Wisconsin, Madison, Wisconsin, United States; 3 Mayo Clinic, Rochester, Minnesota, United States; 4 Michigan Medicine, Ann Arbor, Michigan, United States; 5 University of Illinois at Chicago, Chicago, Illinois, United States

## Abstract

The pandemic has challenged training programs in numerous ways, specifically in the ability to conduct group based teaching sessions. To overcome this challenge Twitter was examined as a vehicle for engaging Geriatric Fellows in education about critical appraisal of clinical research. A secondary objective was to develop educational synergy among university-based programs. To achieve these aims, 5 Midwestern Geriatric and Palliative Medicine Fellowship programs agreed to enroll their fellows into a monthly Geriatrics Twitter Journal Club, that commences on Twitter Tuesday and lasts a week. Each month, an assigned fellow selects an article to discuss and creates a short video to introduce it. A Twitter meister deliveres structured questions to guide fellows’ collective input on the article being critiqued. Over a 3 month roll out of @GeriatricJC, the twitter account of the journal club has gained 144 followers that includes 20 fellows, 63 geriatricians/geriatric faculty, 28 organizational accounts, 5 students and around 28 other providers and experts. From December 2020- February 2021, account generated tweets resulted in an average of 397 impressions/day with 2548 visits to the account profile per month. Videos posted have averaged 73 views/video. Discussion in journal club using #GeriJC has garnered 178 tweets from participants. This project shows that Twitter is a feasible platform for a fellowship journal club among several training programs, thus expanding expertise in evidence-based medicine while lowering the administrative burden of preparing journal club within a single program and increasing both faculty and trainee convenience of learning.

